# Densitometry based microassay for the determination of lipase depolymerizing activity on polyhydroxyalkanoate

**DOI:** 10.1186/2191-0855-3-22

**Published:** 2013-05-08

**Authors:** Diana Hooi-Ean Ch’ng, Kumar Sudesh

**Affiliations:** 1 Ecobiomaterial Research Laboratory, School of Biological Sciences, Universiti Sains Malaysia, Penang, 11800, Malaysia

**Keywords:** Polyhydroxyalkanoate, Lipase, Densitometry, 4-hydroxybutyrate, Hydrolysis

## Abstract

A novel method for the assay of polyhydroxyalkanoate (PHA)-degrading ability of triacylglycerol lipases was developed. By applying the natural affinity of lipases towards hydrophobic interfaces, a sensitive and rapid densitometry analysis for the evaluation of hydrolytic activity of lipase droplets towards PHA-coated surface was successfully carried out. We found that 12 out of 14 tested lipases which are of fungal, bacterial and animal origin were able to hydrolyze P(3HB-*co*-92 mol% 4HB) thin film. The patterns and opacity of the hydrolysis spots of lipases on PHA films allowed easy comparison of PHA-hydrolytic strength of lipases. Lipase from the bacterium *Chromobacterium viscosum* exhibited the highest PHA-degrading activity. The hydrolytic activity of lipases on water insoluble PHA, emulsified *p*-nitrophenyl laurate and olive oil were also compared and interestingly some lipases showed better activity when PHA was used as a substrate.

## Introduction

Polyhydroxyalkanoates (PHAs) are a family of microbial polyesters produced as carbon storage by some bacteria. The most common type of PHA is polyhydroxybutyrate (PHB), which consists of 3-hydroxybutyrate (3HB) monomer units. Besides 3HB, more than 140 different types of monomers have been discovered as the monomer constituents of PHAs ( Steinbüchel 2005 ). Out of the many types of monomers discovered to date, 4-hydroxybutyrate (4HB) provides interesting properties to PHAs. The incorporation of 4HB in a polymer chain containing 3HB results in the production of poly(3-hydroxybutyrate-*co*-4-hydroxybutyrate) [P(3HB-*co*-4HB)] copolymer. The higher the 4HB monomer composition in the copolymer, the more stretchable is the resulting polymer ( Nakamura et al. 1992 ).

Besides its excellent elasticity, P(3HB-*co*-4HB) copolymer also has another advantage which surpasses many other types of PHAs. While the ester bonds between 3HB monomers are known to be naturally hydrolyzed by specific enzymes known as PHB depolymerases, the bondings between 4HB monomers on the other hand are hydrolyzable by the more commonly found triacylglycerol lipases. It is due to the absence of alkyl side chains in the chemical structure of 4HB which renders the straight chain to be a substrate for the enzyme ( Jaeger et al. 1995 ). There are reports showing that eukaryotic lipases were more efficient in degrading P(4HB) homopolymers compared to prokaryotic lipases ( Mukai et al. 1993 ).

The conventional method used for the quantification of 4HB polymer hydrolyzing activity by lipases is very time consuming in order to obtain significant evidence on polymer hydrolysis. It involves weight loss measurement of polymer films incubated in buffered solution of lipases for hours to days, depending on the amount and activity of the enzymes applied ( Ch’ng et al. 2012 ; Saito et al. 1996 ; Vigneswari et al. 2009 ; Jendrossek 2007 ; Jendrossek and Handrick 2002 ). This is because there is a major limitation by using polymer pieces as substrate for enzyme assay, which is low substrate surface area. An efficient assay for enzyme activity requires the enzyme substrate to have as high a surface area as possible in order to increase the sensitivity of the test or the rate of enzymatic reaction. Triglycerides or synthetic carboxylic esters are common substrates used in lipase assay mixture or enzyme activity indicator agar plates. The ability of the substrates to exist in liquid or dissolved form at room temperature has allowed rapid quantification of the release of hydrolysis product or disappearance of substrate during assays ( Hasan et al. 2009 ). The reaction rate of lipases can be further sped up by the addition of emulsifiers to increase the solubility or surface area of the substrate. However, P(3HB-*co*-4HB) copolymers are solid at room temperature regardless of the content of 4HB, and is only soluble in hydrophobic solvent such as chloroform, which will be detrimental to enzyme activity. Besides, due to the rubber-like properties of P(4HB) homopolymer, it is impossible to grind it into powder form, therefore making methods such as preparation of enzyme indicator agar plates not feasible ( Jendrossek 2001 ).

The lack of efficient assays is the major cause for the less extensive study of enzymatic degradation of 4HB-containing PHA compared to P(3HB). In addition, the low yield of the polymer itself is also an important factor that adds to the difficulty in studying polymer degradation ( Volova et al. 2011 ).

Although the hydrolysis of 4HB-containing polymers by lipases was successfully proven by the measurement of polymer weight loss or diameter of clear zones on solution-cast film ( Jaeger et al. 1995 ), a more sensitive method is still needed in order to shed some light on the factors which affect the hydrolysis of 4HB-containing polymers. In earlier studies, the assay of PHA depolymerase activity on PHA film has been investigated by drop assay of the enzyme on thin films of PHA layered on the surface of solid medium ( Schirmer et al. 1993 ). In the present study, a thinly casted film of P(3HB-*co*-4HB) which is transparent was applied for the study of the hydrolysis of the copolymer with high mol fraction of 4HB. The polymer was produced by using the bacterial strain *Delftia acidovorans* (formally known as *Pseudomonas acidovorans*) which is well known for its ability to produce P(3HB-*co*-4HB) copolymer with high 4HB content ( Kimura et al. 1992 ). The method developed in this study requires the incubation of enzyme solution droplets on the surface of the film, and in a matter of minutes opaque hydrolysis spots will be visible on the film surface. The density of the opaque spots can be quantified and correlated to the enzyme concentration. This method provides a more sensitive way to observe and measure the rate of hydrolysis of polymer films by lipases. In addition, comparison with conventional lipase assays revealed that some lipases showed better activity when PHA was used as a substrate.

## Materials and methods

### Biosynthesis of P(3HB-*co*-92 mol%4HB) copolymer

*D. acidovorans* JCM 10181 was cultivated and maintained as previously described ( Lee et al. 2004 ). Two-step cultivation was practiced by using *D. acidovorans* for the biosynthesis of copolymer. For the first step of cultivation, 3% (v/v) of fresh culture was inoculated into 100 mL 2× nutrient broth (NB) medium (pH 7.0) with 1% (w/v) glucose in a 500 mL flask. After 24 h, the cells were harvested by centrifugation at 4000 *g*, 16°C for 10 min. To promote synthesis of PHA, the harvested cells were transferred into the second step medium, which contained 100 mL of nitrogen-free mineral medium (MM) (pH 7.0). The NM consisted of 0.37 g K_2_HPO_4_, 0.58 g KH_2_PO_4_, 0.1 mM MgSO_4_ · 7H_2_O supplemented with 0.1 ml trace elements (TE) solution. 2.78 g FeSO_4_ · 7H_2_O, 1.98 g MnCl_2_ · 4H_2_O, 2.81 g CoSO_4_ · 7H_2_O, 1.67 g CaCl_2_ · 2H_2_O, 0.17 g CuCl_2_ · 2H_2_O and 0.29 g ZnSO_4_ · 7H_2_O (per litre of 0.1 M HCl) were included in the TE solution. 1,4-butanediol was autoclaved separately and added aseptically to the nitrogen-free MM (1% v/v) as the sole carbon source to promote synthesis of the copolymer. Cells were harvested after 48 h of cultivation by centrifugation (4000 *g*, 16°C for 10 min) and washed once with distilled water. Finally, the harvested cells were frozen at -20°C overnight and the frozen cells were then subjected to lyophilization for 48 hours by using a Labconco 4.5 Freezone apparatus.

### Analytical procedures

In order to determine the composition and content of P(3HB-*co*-4HB) copolymer produced by the cells, methanolysis was carried out by the addition of methanolysis solution [15% (v/v) of sulphuric acid and 85% (v/v) of methanol] to about 20 mg of freeze-dried cells and the mixture was heated to 100°C for 140 min. The hydroxyacyl methyl esters then formed were subjected to gas chromatography (GC) analysis ( Braunegg et al. 1978 ) by using a Shimadzu GC-2010 gas chromatograph.

### Extraction and purification of P(3HB-*co*-92 mol% 4HB) copolymer

After the confirmation of PHA composition by GC analysis, the polymer was extracted from the freeze-dried cells. 150 mL of chloroform was added to 1.0 g of the cells and stirred for about 4–5 days at room temperature. Cell debris was then filtered out using a Whatman No.1 filter paper and the collected filtrate was concentrated by using an Eyela rotary evaporator N-1000 until a volume of about 10 mL. A rapidly stirring cold methanol was used to precipitate the PHA from the concentrated filtrate by drop-wise method. Finally, the pure polymer was scooped out from the methanol solution and dried overnight at room temperature.

### Preparation of thin PHA film

20 mg of P(3HB-*co*-92 mol% 4HB) polymer was dissolved in 20 mL of chloroform by stirring for about 15 minutes at room temperature (25°C). The polymer solution was poured into a 9 cm glass petri dish and covered with perforated aluminium foil. The solvent was allowed to evaporate by leaving the petri dish on a well balanced surface (balanced by using a bubble level) to prevent unequal thickness of film. The transparent polymer film formed on the dish (approximately 4 μm of thickness) was aged for 3 days and used directly for enzyme assay without peeling off from the dish.

### Qualitative observation of PHA film hydrolysis by lipases

Lyophilized powder of 14 lipases (Sigma Aldrich) (Table 1) was dissolved in phosphate buffer solution (100 mM, pH7.4) to the desired concentration in 1.5 mL Eppendorf tubes and vortexed until they are completely dissolved. All lipase preparations were used without further purification. 20 μL of lipase solution was pipetted onto the polymer film surface to form semispherical droplets. Markings were made at the bottom surface of the glass petri dish by a marker pen prior to dropping to assist in the location of droplets and separation of samples and controls. The polymer dish with droplets was then covered with aluminium foil and incubated at 37°C for 30 min. After incubation, the film was immediately flooded with distilled water to wash away the enzyme droplets. The film was then allowed to dry at room temperature before observations were recorded.

**Table 1:** List of commercial lipases used in the current study (Sigma-Aldrich technical information)

**Lipase name**	**Product number**	**Lipase activity (U/mg)**	**Unit definition**
*Lipase from Aspergillus oryzae*	62285	~50	1 U corresponds to the amount of enzyme which liberates 1 μmol oleic acid per minute at pH 8.0 and 40°C (triolein, Fluka No. 62314 as substrate)
*Amano Lipase A from Aspergillus niger*	534781	≥12	-
*Lipase from Candida antarctica*	65986	≥1.0	1 U corresponds to the amount of enzyme which liberates 1 μmol oleic acid per minute at pH 8.0 and 40°C (triolein, Fluka No. 62314 as substrate)
*Lipase from Candida rugosa, Type VII*	L1754	≥700	1 U will hydrolyze 1.0 microequivalent of fatty acid from a triglyceride in 1 hr at pH 7.2 at 37°C
*Amano Lipase M from Mucor javanicus*	534803	≥10	-
*Lipase from Mucor miehei*	L9031	≥4,000	1 U will hydrolyze 1.0 microequivalent of fatty acid from a triglyceride in 1 hr at pH 7.7 at 37°C using olive oil
*Lipase from Rhizopus arrhizus*	62305	~10	1 U corresponds to the amount of enzyme which liberates 1 μmol of butyric acid per minute at pH 8.0 and 40°C (tributyrin, Fluka No. 91010 as substrate) 5000 U as described above are equivalent to ~1 U using triolein, Fluka No. 62314 as substrate, at pH 8.0 and 40°C
Lipase from *Rhizopus niveus*	62310	≥1.5	1 U corresponds to the amount of enzyme which liberates 1 μmol fatty acid from a triglyceride per minute at pH 7.7 and 40°C (olive oil as substrate)]; 300 U as described above are equivalent to ~1 U using triolein, Fluka No. 62314, at pH 8.0 and 40°C as substrate
Lipase from *Rhizopus oryzae*	80612	≥30	1 U corresponds to the amount of enzyme which releases 1 μmol fatty acid from triglycerides per minute at pH 7.2 and 37 C (olive oil as substrate)
*Lipase from Chromobacterium viscosum*, Type XII	L0763	≥2,000	1 U will hydrolyze 1.0 microequivalent of fatty acid from a triglyceride in 1 hr at pH 7.7 at 37°C using olive oil (30 minute incubation)
*Lipase from Pseudomonas cepacia*	62309	≥30	1 U corresponds to the amount of enzyme which liberates 1 μmol oleic acid per minute at pH 8.0 and 40°C (triolein, Fluka No. 62314 as substrate)
*Amano Lipase from Pseudomonas fluorescens*	534730	≥20	-
Lipase from porcine pancreas, Type II	L3126	100-400 (using olive oil, 30 min incubation), 30–90 (using triacetin)	1 U will hydrolyze 1.0 microequivalent of fatty acid from triacetin in 1 hr at pH 7.4 at 37°C. (pH 7.7 is used with olive oil as substrate)
Lipase from wheat germ, Type I	L3001	5-15	1 U will hydrolyze 1.0 microequivalent of fatty acid from a triglyceride in 1 hr at pH 7.7 at 37°C

### Quantitative analysis of hydrolysis spot density

A scanned picture of the hydrolysis spots on polymer film was obtained by placing the petri dish facing upwards on a CanoScan LiDE 20 paper scanner. For the quantification of hydrolysis spot intensity, the dish was scanned by placing a piece of black cardboard at the back of it with the lid of the scanner left open to obtain a JPEG image (2480×3507 pixels) with a completely dark background. The contrast of the picture was optimized by using picture editing software. 90×90 pixels square images beneath an enzyme droplet or a control droplet were then cropped from the picture. The square images were pasted on an empty slide of Microsoft Office PowerPoint and arranged in a row with the control spot image positioned at leftmost. The arranged images were then selected and saved as a new picture (JPEG file). The picture was then color-inverted and subjected to densitometry analysis by using the ImageJ densitometry software (Version 1.44, NIH). An area of w (width) = 40 and h (height) = 40 was selected on the control square by using the ‘rectangular’ tool in the software. The control image was selected as ‘first lane’ and subsequent sample images were selected as ‘next lane’. Finally, ‘plot lanes’ command was selected to obtain density plots for each square image.

Next, the entire area beneath each plot line was highlighted by using the ‘straight’ tool in the software. Then the ‘Wand (tracing) tool’ was selected and by clicking the cursor at the highlighted area beneath each plot line, the area of each plot was obtained. These values were used to calculate the relative density on each square image by using the formula:

$$\begin{array}{c} \mathrm{Relative}\;\mathrm{density}\;\mathrm{of}\;\mathrm{hydrolysis}\;\mathrm{spot}\\ \;=\frac{\mathrm{Plot}\;\mathrm{area}\;\mathrm{of}\;\mathrm{hydrolysis}\;\mathrm{spot}}{\mathrm{Plot}\;\mathrm{area}\;\mathrm{of}\;\mathrm{control}\;\mathrm{spot}} \end{array}$$

In addition, the specific activity of each lipase was also calculated as shown below:

$$\begin{array}{c} \mathrm{Specific}\;\mathrm{PHA}-\mathrm{hydrolyzing}\;\mathrm{activity}\\ \;=\frac{\mathrm{Relative}\;\mathrm{density}}{\mathrm{Incubation}\;\mathrm{time}\;\left(\min\right)\times \mathrm{weight}\;\mathrm{of}\;\mathrm{enzyme}\;\mathrm{in}\;\mathrm{droplet}\;\left(\mathrm{mg}\right)*} \end{array}$$

* Weight of enzyme in droplet = Concentration of enzyme solution (mg/mL) × Volume of droplet (mL)

### Modeling the effect of lipase concentration on relative density of hydrolysis spots

Hydrolysis spot density was plotted against lipase concentration to study the trend of spot density along a range of different enzyme concentrations. The curve obtained was fitted with various mathematical models and the model which yielded the coefficient of determination (R^2^) that is closest to 1 was selected.

### Lipase activity assay with fatty acid ester

The measurement of hydrolytic activity of lipases on fatty acid esters was carried out with some modifications on the method of Kilcawley and coworkers ( Kilcawley et al. 2002 ). Lipase activity was measured spectrophotometrically with the use of *p*-nitrophenyl laurate (*p*NPL) as enzyme substrate. 5 mM of *p*NPL was dissolved in 10 mL of dimethyl sulphoxide (DMSO). The solution was then emulsified in 90 mL of phosphate buffer (100 mM, pH 7.4) which contains 0.1% (w/v) polyvinyl alcohol (PVA) and 0.4% (w/v) Triton X-100 to form the assay mixture. A 100 μL of lipase solution was mixed with 1.9 mL of the assay mixture and incubated at 37°C for 10 min. The final absorbance of the mixture was measured at 410 nm by a Jenway 6505 UV/Vis spectrophotometer. The amount of 4-nitrophenol released was calculated by using a standard curve of 4-nitrophenol concentration versus absorbance. The following equation was used to obtain the specific activity of each lipase with *p*NPL as substrate:

$$\begin{array}{c} \mathrm{Specific}\;p\mathrm{NPL}-\mathrm{hydrolyzing}\;\mathrm{activity}\\ \;=\frac{\mathrm{Amount}\;\mathrm{of}\;4-\mathrm{nitrophenol}\;\mathrm{released}\left(\mathrm{\mu mol}\right)}{\mathrm{Incubation}\;\mathrm{time}\;\left(\min\right)\times \mathrm{weight}\;\mathrm{of}\;\mathrm{enzyme}\;\mathrm{in}\;\mathrm{assay}\;\mathrm{mixture}\;\left(\mathrm{mg}\right)†} \end{array}$$

† Weight of enzyme in assay mixture = Concentration of enzyme solution (mg/ mL) × Volume of enzyme solution added into assay mixture (mL)

### Lipase activity assay with triglyceride

Lipase assay by using olive oil as substrate was carried out and the activity was determined by measuring the free fatty acid (FFA) released ( Kwon and Rhee 1986 ). The total assay mixture consisted of 1.0 mL lipase solution, 2.5 mL olive oil emulsion and 0.02 mL of 20 mM CaCl_2_ · 2H_2_O. The olive emulsion was prepared by vigorous stirring of olive oil (Bertolli, Italy) and 50 mM PBS (pH7.4) in the ratio of 1:1 until whitish and milky. Samples containing the assay mixture were incubated for 30 min with shaking at 200 rpm at 37°C. Reaction was stopped at the end of incubation by the addition of 1.0 mL of 6N HCl and 5.0 mL of isooctane followed by vortexing. Control samples were prepared by adding HCl and isooctane immediately into the assay mixture without incubation. The upper isooctane layer (3.0 mL) containing FFA was transferred into a test tube, and 1.0 mL of copper reagent was added and vortexed. FFA will then form complex with the copper reagent to form cupric soap (blue color) that is measurable at a wavelength of 715nm ( Pinsirodom and Parkin 2001 ). Copper reagent was prepared by preparing an aqueous solution of copper (II) acetate monohydrate (5% *w/v*) and adjusting the pH to 6.1 by using pyridine. Upon the settling of the isooctane-copper pyridine mixture into two distinct layers, 2 mL of the upper layer were subjected to absorbance measurement at 715 nm by using isooctane as blank. The amount of FFA released was determined from a standard curve of oleic acid concentration versus absorbance.

## Results

### Screening of lipases with the ability to hydrolyze P(3HB-*co*-92 mol% 4HB) film

14 commercial lipases were screened for their ability to hydrolyze thin cast film of P(3HB-*co*-92 mol% 4HB). All lipases (1 mg/mL) that were able to leave a visible mark on the polymer surface after incubation were considered to show positive results in polymer hydrolysis. The results were compared to the control (bovine serum albumin) which did not cause any visible changes to the film surface.

Table 2 lists the lipases that were used in this study and their respective polymer hydrolysis results. Out of the 14 lipases tested, 12 lipases showed positive signs of polymer hydrolysis. Negative results were obtained for lipases from *A. niger* and wheat germ.

**Table 2:** **Types of lipases and their ability to hydrolyze thin films of P(3HB- *co *-92 mol% 4HB)**

**Types of lipases**	**Sources of lipases**	**Hydrolytic ability on P(3HB- *co *-92 mol% 4HB) film**
Fungal	*Aspergillus oryzae*	Positive
	*Aspergillus niger*	Negative
	*Candida antarctica*	Positive
	*Candida rugosa*	Positive
	*Mucor javanicus*	Positive
	*Mucor miehei*	Positive
	*Rhizopus arrhizus*	Positive
	*Rhizopus niveus*	Positive
	*Rhizopus oryzae*	Positive
Bacterial	*Chromobacterium viscosum*	Positive
	*Pseudomonas cepacia*	Positive
	*Pseudomonas fluorescens*	Positive
Animal	Porcine pancreas	Positive
Plant	Wheat germ	Negative

### Effect of lipase concentration on hydrolysis patterns

Initially, 4 types of lipases were selected to study the effect of lipase concentration on hydrolysis patterns. The lipase solutions were serial diluted 2 times to obtain 3 different concentrations and were dropped on the PHA film and incubated.

**Figure 1:**
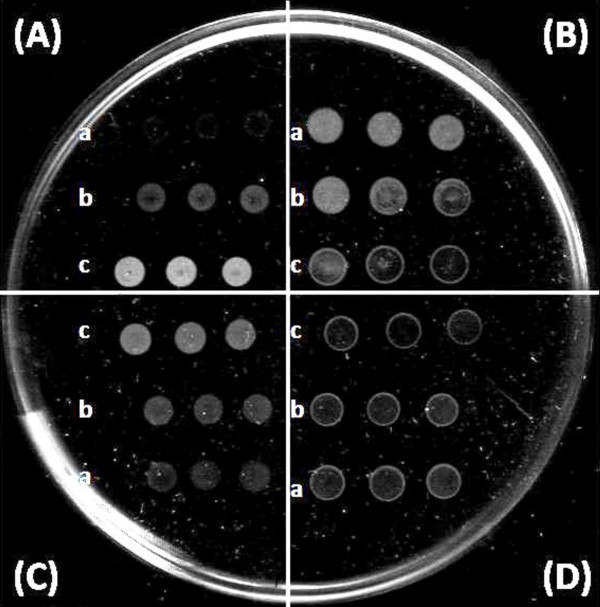
**Effect of lipase concentration on hydrolysis patterns. (A)***P. cepacia* lipase, **(B) ***R. arrhizus* lipase, **(C)***R. oryzae *lipase, and **(D)***A. oryzae* lipase. (a) 0.125 mg/mL, (b) 0.25 mg/mL, (c) 0.5 mg/mL. Each row with 3 adjacent spots indicated triplicates.

Figure 1 shows the scanned picture of PHA film after incubation with different concentrations of lipase solutions. Although it can be observed that all the tested lipases hydrolyzed the surface of the polymer film and produced visible round spots when dropped onto the film, the intensities of hydrolysis spots changed when the lipase concentration was varied. Different trends of hydrolysis spot intensities can be observed for lipases from *P. cepacia*, *R. arrhizus* and *R. oryzae*. For the lipases from *P. cepacia* and *R. arrhizus*, the intensity of opaque spots decreased as the enzyme concentration decreased. On the other hand, the lipase from R. oryzae showed an opposite trend, whereby its hydrolysis spot intensity increased as the enzyme concentration was decreased. Interestingly, the lipase from *A. oryzae* produced ring patterns on PHA film regardless of the enzyme concentration. Besides detecting the presence of depolymerizing activity, the PHA film used can also differentiate lipases with different concentrations by producing hydrolysis spots with a variety of opacity. Therefore, these enzymes together with the other lipases which were shown to be able to hydrolyze thin P(3HB-*co*-92 mol% 4HB) films were selected to further investigate the effect of enzyme concentration on hydrolysis spot intensities by using a bigger range of enzyme concentrations.

### Concentration profile of polymer hydrolysis

Different concentrations of a lipase produced opaque spots with different patterns and intensities on polymer films. Therefore, a concentration profile for the opaque spot intensities could be constructed to further investigate the trend for the hydrolysis spots.

**Figure 2:**
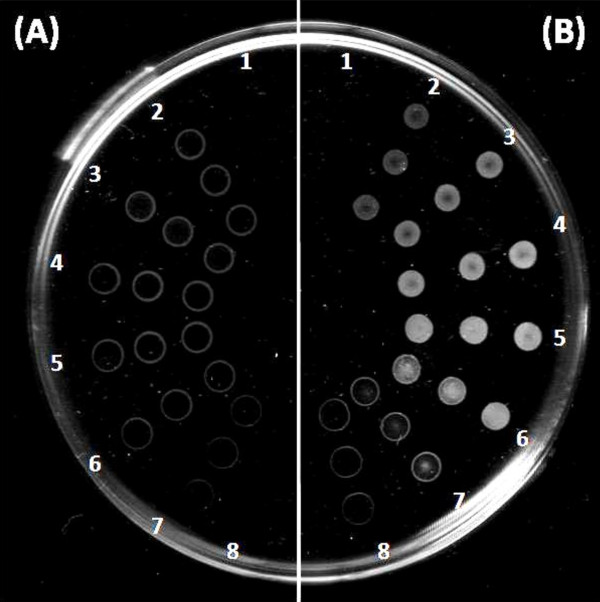
**Concentration profiles of polymer hydrolysis. (A) ***A. oryzae* lipase, **(B)***P. cepacia* lipase. The concentrations of lipases increase from number 2 (0.03125 mg/mL) to number 8 (2 mg/mL). Number 1 shows control without enzyme. Each row with 3 adjacent spots indicates triplicates.

Figure 2 shows a scanned picture of hydrolysis spots left by the lipases from *A. oryzae* (A) and *P. cepacia* (B) over a range of concentrations. These 2 lipases were selected as representatives because the patterns observed for all 12 types of lipases tested can be grouped into those as shown by the 2 model lipases. In order to obtain the patterns as shown in Figure 2, the enzyme solutions were serial diluted from a stock with a concentration of 2 mg/mL and dropped onto the film surface. The hydrolysis spots created by *P. cepacia* lipase portrayed the typical trend of most lipases. The intensity of the opaque spots increased gradually from faint to intense with the increase of lipase concentration, until a stage when the rate was fast enough to cause complete hydrolysis of the film under the droplets (substrate depletion). At this stage the opaque spot gradually disappeared and revealed the transparent part of the glass petri dish beneath it.

The spots produced by *A. oryzae* lipase portrayed an unusual pattern whereby the lipase only hydrolyzed the edges of the droplet (Figure 2A). The change in enzyme concentration did not show obvious effect on the intensity of the ring patterns produced. Only qualitative observation of polymer hydrolysis was possible with this type of lipase.

**Figure 3:**
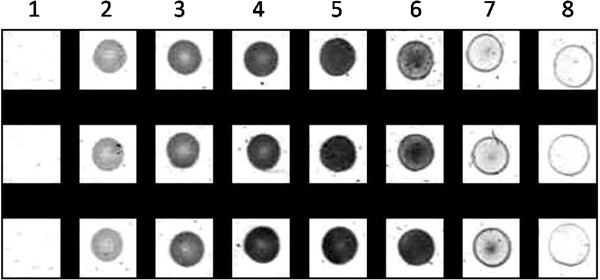
**Cropped, aligned and color-inverted picture of hydrolysis spots by *P. cepacia *lipase from low (2) to high concentration (8).** Each column shows triplicates. Column (1) shows control without enzyme.

In order to quantify the degree of enzymatic degradation on the polymer film, the scanned photograph of the spots on the polymer dish was used for densitometry software analysis. Figure 3 shows a picture of the scanned, cropped, aligned and color-inverted version of the hydrolysis spots by *P. cepacia* lipase. Opaque spots appeared white on the polymer film surface but the scanned picture was inverted to make the spots look dark on a white background. This is to allow the density of the spots to be analyzed by densitometry software.

After densitometry analysis of the spots, the relative density of the hydrolysis spots to control can be obtained. A plot of average relative density of hydrolysis spots versus concentration was plotted in order to obtain a model for the effect of enzyme concentration on the density of hydrolysis spots on the polymer film.

**Figure 4:**
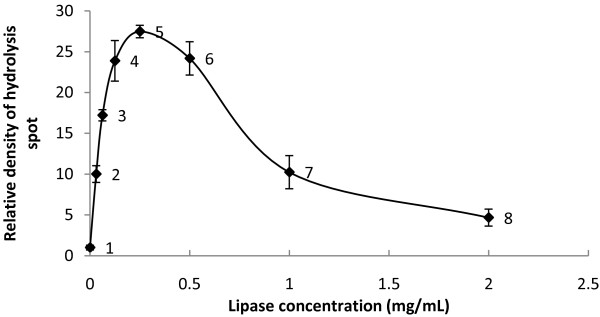
**Plot of relative density of hydrolysis spots versus concentration of *P. cepacia *lipase.** Points 1–8 correspond to the spots on Figure 3

Figure 4 shows the plot of relative density of hydrolysis spots versus lipase concentration of *P. cepacia*. The points 1 to 8 were obtained by the average relative density of the spots shown in Figure 3. Point number 1 to 5 showed a gradual increase of relative intensity from low to maximum. At lower enzyme concentrations, the substrate was not saturated with enzymes and therefore the rate of reaction increases proportionally with the increase of enzyme concentration. When the substrate becomes saturated with enzymes, further increase in the amount of enzyme did not cause significant increase to the enzymatic rate. Point number 6 to 8 showed a gradual decrease of relative intensity when the enzyme concentration was high enough to lyze through the polymer film. This type of observation indicated the occurrence of substrate depletion.

### Model of effect of lipase concentration on relative density of hydrolysis spots

In order to study the relationship between the relative density of hydrolysis spots and enzyme concentration, a plot by using only the range of concentrations before the occurrence of substrate depletion was constructed. For the lipase of *P. cepacia*, point 1 to 5 in Figure 4 would represent the range where the rate of enzymatic reaction is affected by the enzyme concentration.

**Figure 5:**
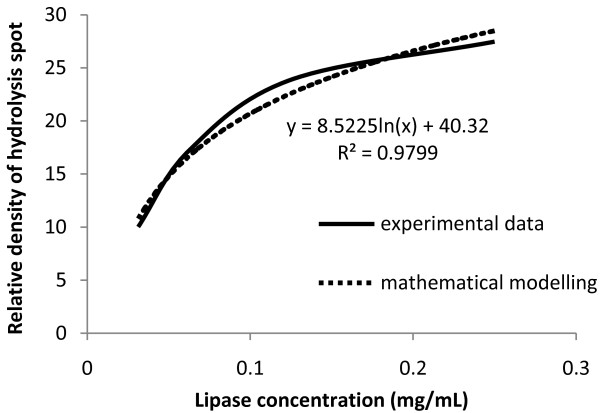
**Model for the effect of lipase concentration on relative density of hydrolysis spots by *P. cepacia *lipase.**

Figure 5 shows the modeling of the relationship between the densities of hydrolysis spot to lipase concentration of *P. cepacia*. The plot generates the shape of a saturation curve, which is common for many biological and enzymatic reactions. The steepness of the curve is higher at the initial part of the plot (higher initial rate of reaction) and becomes less steep as the concentration increases further (reaction rate continuously slowed down). This indicated the occurrence of substrate saturation. Apart from the lipase from *A. oryzae*, this shape of curve was obtained for the rest of the tested lipases (results not shown). This shows that it is possible to use this method to construct a standard curve for the estimation of lipase solutions with unknown concentrations by measuring the intensity of its hydrolysis spot on polymer film.

### Range of concentrations of lipases before substrate depletion and their relationship to polymer hydrolytic strength

In order to use thin polymer films for enzyme concentration estimations, it is crucial to obtain the range of concentrations for the lipases which are able to be detected by the film. This is because once the hydrolysis rate is too high; the opacity of the hydrolysis spots will be lost and no longer be proportional to the enzyme concentration.

**Figure 6:**
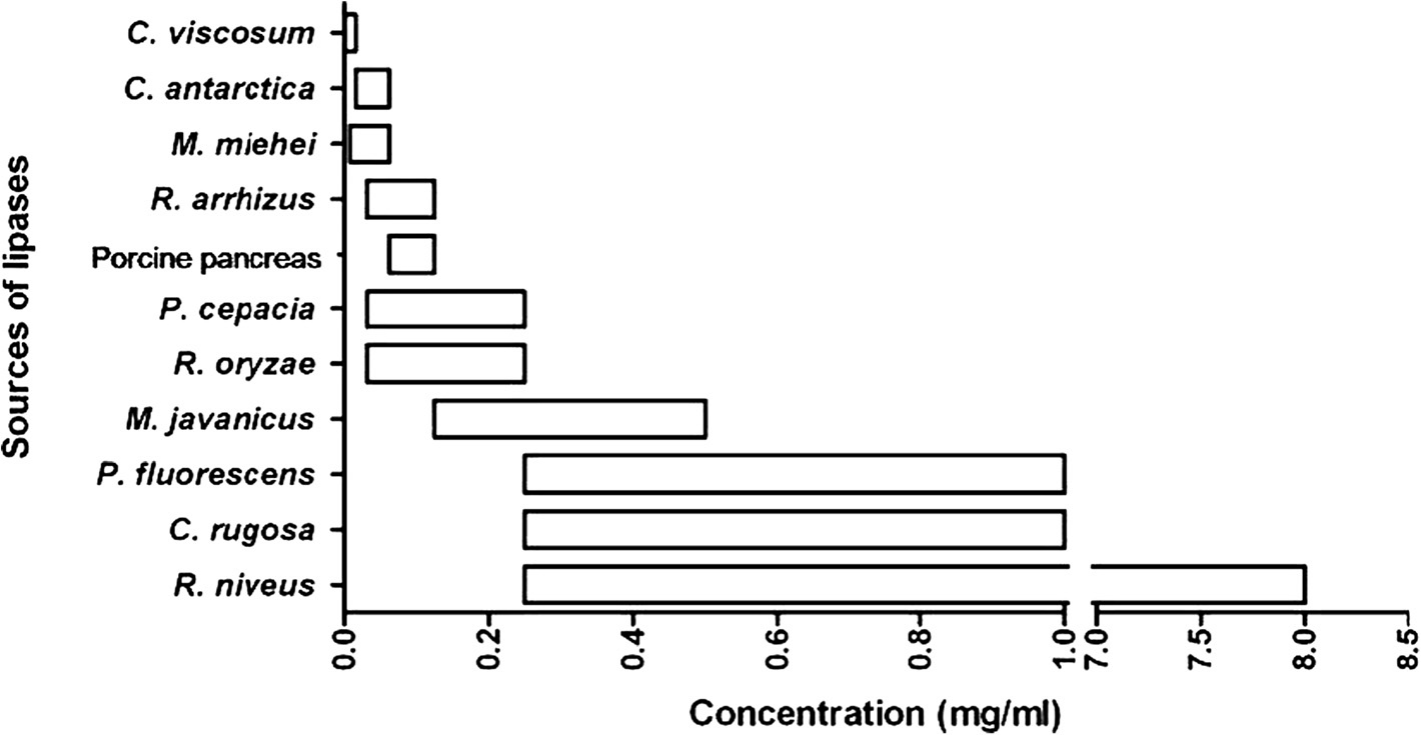
**Range of concentration of different lipases with measurable opacity by using thin P(3HB-*co*-92 mol% 4HB) film.**

Figure 6 shows the range of concentrations for 11 lipases which were able to produce measurable opacity on the casted film surface. The lower limit of the range indicated the minimum concentration of the lipase that can produce visible spot on the film whereas the higher limit represents the concentration for maximum opacity before substrate depletion. Besides the lipase from *R. niveus*, a majority of lipases studied caused substrate depletion within the range of 0.01 to 1 mg/mL. This shows that the rate of enzymatic degradation of lipase from *R. niveus* was much slower if compared to the other enzymes since a concentration as high as 8 mg/mL was required to achieve substrate depletion. On the other hand, lipase from *C. viscosum* showed exceptionally high hydrolytic activity towards the polymer film (substrate depletion at 0.01 mg/mL). It is however notable that the range hereby reported is only applicable to the thickness of film as casted in this study. A thicker film will support higher concentrations of lipases or longer incubation period before substrate depletion occurs.

### Comparison of lipase specific activities on assays with PHA film, emulsified *p*NPL and olive oil as substrates

The specific hydrolytic activity of lipases was compared by using the polymer film, emulsified *p*NPL, as well as olive oil as substrate. For the assay of PHA-hydrolyzing ability, all lipase solutions were diluted to a concentration, which is within the ranges shown in Figure 6 before being dropped onto the polymer cast film. The film was then incubated prior to the analysis with densitometry software. The assay with *p*NPL and olive oil was carried out as mentioned in the Materials and methods. Apart from the bacterial lipase from *C. viscosum*, which was ranked first in all 3 assays, all the other tested lipases performed differently in each assay. Our results showed that 3 out of 12 lipases tested preferred the PHA film as substrate. The eukaryotic lipases from *C. antarctica, R. oryzae* and porcine pancreas showed higher rankings in the assay with PHA compared to that with *p*NPL and olive oil (Table 3). In contrast, all 3 of the prokaryotic lipases (*C. viscosum, P. cepacia and P. fluorescens*) showed higher rankings when assayed with the method of *p*NPL and olive oil. It is interesting to note that the lipase from *C. antarctica* which was ranked second in the assay with PHA film was ranked last when assayed by using olive oil as substrate.

**Table 3:** **Specific activities and ranking of lipases in P(3HB- *co *-92 mol% 4HB), *p*NPL and olive oil hydrolysis assay**

**Sources of lipases**	**P(3HB- *co *-92 mol% 4HB)**		***p*NPL**		**Olive oil**	
	**Specific activity (Relative density/ min/ mg)**	Rank*	**Specific activity (μmol 4-nitrophenol/ min/ mg)**	**Rank†**	**Specific activity (μmol fatty acid/ min/ mg)**	**Rank**^**#**^
*C. viscosum*	4369.47	1	9800	1	365.00	1
*C. antarctica*	596.29	2	44	4	0.60	11
*M. miehei*	249.67	3	8.1	6	49.35	2
*P. cepacia*	58.76	4	80	2	15.28	3
*R. oryzae*	52.8	5	0.1	10	6.19	6
*R. arrhizus*	45.52	6	6.2	7	10.69	4
Porcine pancreas	32.19	7	0.25	8	0.90	10
*P. fluorescens*	23.97	8	37	5	5.64	7
*M. javanicus*	21.05	9	0.15	9	2.83	8
*C. rugosa*	15.21	10	68	3	8.72	5
*R. niveus*	1.06	11	0.03	11	1.53	9

* Rank according to specific activity by using P(3HB-*co*-4HB) as substrate, †Rank according to specific activity by using *p*-NPL as substrate, ^#^Rank according to specific activity by using olive oil as substrate.

## Discussion

In the present study, 12 out of 14 lipases tested showed positive signs of polymer degradation by using the novel microassay without the addition of emulsifiers or other supplements (Table 2). A small droplet of concentrated lipase solution was sufficient to show very convincing evidence of polymer degradation (opaque marks) in a matter of minutes. The work of Timmins and coworkers ( Timmins et al. 1997 ) further supports the idea of increasing opacity of transparent polymer film by the action of depolymerase enzymes. This may be due to the enzyme acting first on the amorphous part of the film, which leaves out the crystalline portion which is more opaque ( Kumagai et al. 1992 ; Sudesh et al. 2000 ). It may also be simply due to the corrosion of the smooth surface of the polymer film by the action of enzymes, which affected the light scattering property of the film, causing it to lose transparency.

Not all types of lipases tested in this study showed positive signs of polymer degradation with this method. The physical force which inhibits the action of the lipase from *A. niger* and wheat germ from acting on the polymer film is still unknown. The observation of the lipase from *A. oryzae*, which hydrolyzed only the edge of the contact area of water and polymer (ring pattern), suggested that some enzymes may be capable of hydrolyzing the polymer film, however, an unknown inhibiting factor had forced the enzymes away from the solid–liquid interface. It has been reported that some lipases can be denatured under high interfacial tension. Reis and coworkers ( Reis et al. 2009 ) have highlighted the importance of interfacial properties in affecting the activation of lipase molecules and this factor may be playing a major role in determining the ability of lipases to hydrolyze PHA films.

A careful observation of the hydrolysis spots in Figure 3 also revealed the fact that the opacity of hydrolysis spots was not always equally distributed throughout the circular boundary. At lower enzyme concentration, the intensity was seen to be highest at the boundary of the enzyme droplets, and decreased towards the center of the spot. When the film surface was saturated with enzymes, the spot became fully opaque. As the enzyme concentration increased further, substrate depletion occurred. This process also started with the loss of opacity at the boundary of the spot first, and continued towards the middle part of the spot. The observation of non-equal rate of hydrolysis under a droplet was also found when lipases were dropped on tributyrin agar plates ( Lee et al. 2012 ). This shows that the pattern is common for the action of lipases on hydrophobic surfaces.

Due to the observation of ring or full opaque spot patterns, and high or low intensity of opaque spots produced by different types of lipases, it was initially thought that each type of lipase may produce their own unique patterns on polymer films. After further investigation of the effect of lipase concentration on hydrolysis spot patterns, it was revealed that the patterns were actually caused by different reaction rate of the enzymes. Different lipases may produce the same spot intensity at different concentration. It is then very obvious that this novel assay method not only detects the hydrolysis of polymer film by lipases, but also differentiates the rate of reaction or the activity of the enzymes on the film.

A concentration profile for all 12 types of lipases were constructed by dropping different concentrations of the same type of enzyme on a single film and observing the patterns and intensity of opaque spots produced after incubation. Apart from the lipase of *A. oryzae*, which produced ring patterns regardless of its concentration, the other 11 types of lipases all showed a similar trend of increasing opacity when lipase concentration was increased (results not shown). In order to obtain a nice concentration profile for each lipase, its minimum and maximum concentration that can produce opaque spots visible to the naked eye should be taken into consideration. The minimum concentration marks the point where the lowest hydrolytic activity on the film can be observed whereas the maximum concentration is the point before the rate of hydrolysis is fast enough to cause substrate depletion. It is difficult to differentiate between no hydrolysis and substrate depletion, since both also leave behind a transparent background. A very simple and effective method for the confirmation of substrate depletion is by scraping the surface of the hydrolysis spot with something pointed such as the tip of a mechanical pencil. If there is no substrate depletion, there will be a scrape mark produced, showing that there are still traces of polymer left on the glass petri dish. Similarly, if the thin polymer film had been hydrolyzed into water soluble subunits, and are washed away during the rinsing step after incubation with lipase solution, then it will be impossible to produce a scrape mark on the petri dish. The different working ranges of the film in detecting polymer degrading ability of the lipases are shown in Figure 6. It is important to bear in mind that with different thickness of cast film produced, incubation time, or lipase concentration, the range may shift depending on the enzymatic rate and substrate amount beneath the lipase droplet.

In order to measure the rate of enzymatic activity of each lipase, a quantitative value of the hydrolysis spots must be obtained. This can be achieved by applying the theory of reading the intensity of gel electrophoresis bands by using densitometry analysis software. The feasibility of the method was proven since the intensity of hydrolysis spots measured by the software was shown to increase proportionally to the enzyme concentration or enzymatic rate (Figures 4 and 5).

In comparison to the common liquid-liquid phase lipase assays such as titrimetric, colorimetric and spectrophotometric assays ( Beisson et al. 2000 ), the current solid–liquid phase densitometric assay has the advantage of being easily conducted with minimal time for assay medium preparation. Besides, there is an added advantage on the easy recovery of hydrolysis end product or enzymes for further analysis after each reaction. Since PHA film (enzyme substrate) is used in its solid form throughout the assay, there is little difficulty in the separation of substrate and end product. The method is also useful for the assay of enzymes which are available in low quantity because only minute amount of the proteins is needed and the enzymes may be recovered after the assay.

Although the discovery of the ability of lipases to degrade PHA dates back to the 1970’s ( Tokiwa and Suzuki 1977 ), this substrate has not been used widely by researchers who are studying lipases. However, from the results in this study, PHA with high 4HB content looks like a very promising substrate for the study of hydrolytic activity of lipases on polymers. Table 3 shows the comparison of lipase assay results obtained by using P(3HB-*co*-92 mol% 4HB) film, *p*NPL and olive oil as enzyme substrates respectively. *p*NPL is the synthetic carboxylic ester of 4-nitrophenol with lauric acid, whereas olive oil is commonly used to assay for lipase activity on its native substrate, which is triglyceride. The purpose of the comparison is to screen for lipases which have a better activity with the PHA hydrolytic microassay compared to the assay with the commonly used substrate. By comparing the specific activity ranking of lipases in all three assays (Table 3), some of the eukaryotic lipases tested in this study, which are lipases from *C. antarctica, R. oryzae* and porcine pancreas were shown to have better activity in hydrolyzing the PHA film. This observation generally corresponded well with previous reports which mentioned that eukaryotic lipases have better PHA-degrading ability ( Jaeger et al. 1995 ; Mukai et al. 1993 ). However, as opposed to previous study suggesting that lipases from the genus *Rhizopus* have the highest activity in degrading polymers ( Mukai et al. 1993 ; Tokiwa and Suzuki 1977 ), the lipase from *R. oryzae* was ranked number 5 in this study, which was preceded by 4 other lipases of prokaryotic and eukaryotic sources. In order to have a more valid comparison of depolymerizing-activity between lipases, the purity of the enzyme preparation has to be taken into account.

It was reported previously by Jaeger et al. ( Jaeger et al. 1995 ) that the depolymerase activity of *P. fluorescens* lipase (0.025 mg/mL) was not detected when tested on P(4HB) substrate. According to the results obtained from this study, aqueous solution of *P. fluorescens* lipase can only show visible degradation on PHA film when its concentration was above 0.25 mg/mL (Figure 6). Therefore, a more concentrated solution of lipases are required to observe their depolymerase activity on 4HB-containing polymer. The availability of commercialized lipases had allowed the screening of lipase preparations with high concentrations and the ability of prokaryotic lipases to hydrolyze PHA was further confirmed in this study. Although they have better performances in the conventional lipase assays (Table 3), prokaryotic lipases also have significant activity in the PHA-hydrolyzing assay, with lipase from *C. viscosum* leading in all 3 assays methods. Altogether, the results have proven the applicability of the current assay in detecting PHA-degrading ability of lipases of eukaryotic and prokaryotic sources. The method is much anticipated to be applied in the screening of PHA-degrading enzymes of human and animal origin, and also for the development of high-throughput screening of lipase activity.

In conclusion, a novel method for sensitive detection and quantification of PHA degrading ability of triglyceride lipases has been developed in this study. 12 out of 14 lipases from fungal, bacterial and animal sources were shown to be able to degrade P(3HB-*co*-92 mol% 4HB) thin film via densitometric analysis. When the specific activities of lipases which were assayed by using PHA, *p*NPL and olive oil as substrates were compared, 3 lipases which are of eukaryotic sources showed higher preferences on the polymer film. In addition, prokaryotic lipases were also shown to have significant ability to degrade PHA. The current microassay has opened up a new application for the use of P(3HB-*co*-4HB) copolymer as a substrate for detecting and quantifying lipase activity.

## Competing interests

DHEC and KS have filed for patent application for the method to assay PHA-degrading activity of triacylglycerol lipases described in this paper.

## Authors’ contributions

DHEC and KS participated in the design of the experiment. DHEC performed all the experiments. DHEC and KS analyzed the data. DHEC wrote the manuscript. KS edited the manuscript. All authors read and approved the final manuscript.
